# Surgical management of a splenic artery aneurysm

**DOI:** 10.1002/ccr3.550

**Published:** 2016-03-29

**Authors:** Kamuran Cumhur Değer, Ebubekir Gündeş, Ali Fedakar

**Affiliations:** ^1^ Gastroenterological Surgery DepartmentKartal Koşuyolu High Speciality and Training HospitalIstanbulTurkey; ^2^ Cardiovascular Surgery DepartmentKartal Koşuyolu High Speciality and Training HospitalIstanbulTurkey

**Keywords:** Anastomosis, aneurysm, angioembolization, splenic artery

## Abstract

Aneurisms of the splenic artery are rare clinical findings. Surgeons and interventional radiologists should co‐operate in the management of this challenging disease; we describe here a surgical option.

**Question 1**: What is your diagnosis when you look at the CT angiographic slice of the abdomen?

**Answer 1**: The physicians at our cardiology clinic referred a patient (Z.D/prot:127, 77/F) to us, who had been suffering from epigastric pain for 3 months and was diagnosed with splenic artery aneurism (SAA) via ultrasonography and CT angiographic imaging. A 37 × 38 mm SAA was revealed in the middle portion of the splenic artery, beneath the posterior pancreatic corpus (Fig. 1). The aneurysm was not suitable for angioembolization due to its size and arterial configuration. The most catastrophic event in the course of these patients is the rupture of the aneurysm, and the most important risk factor is whether the aneurysm is greater than 2 cm 1. Because the patient was symptomatic and the aneurysm size was larger than 2 cm, we decided to perform surgery.

**Figure 1:**
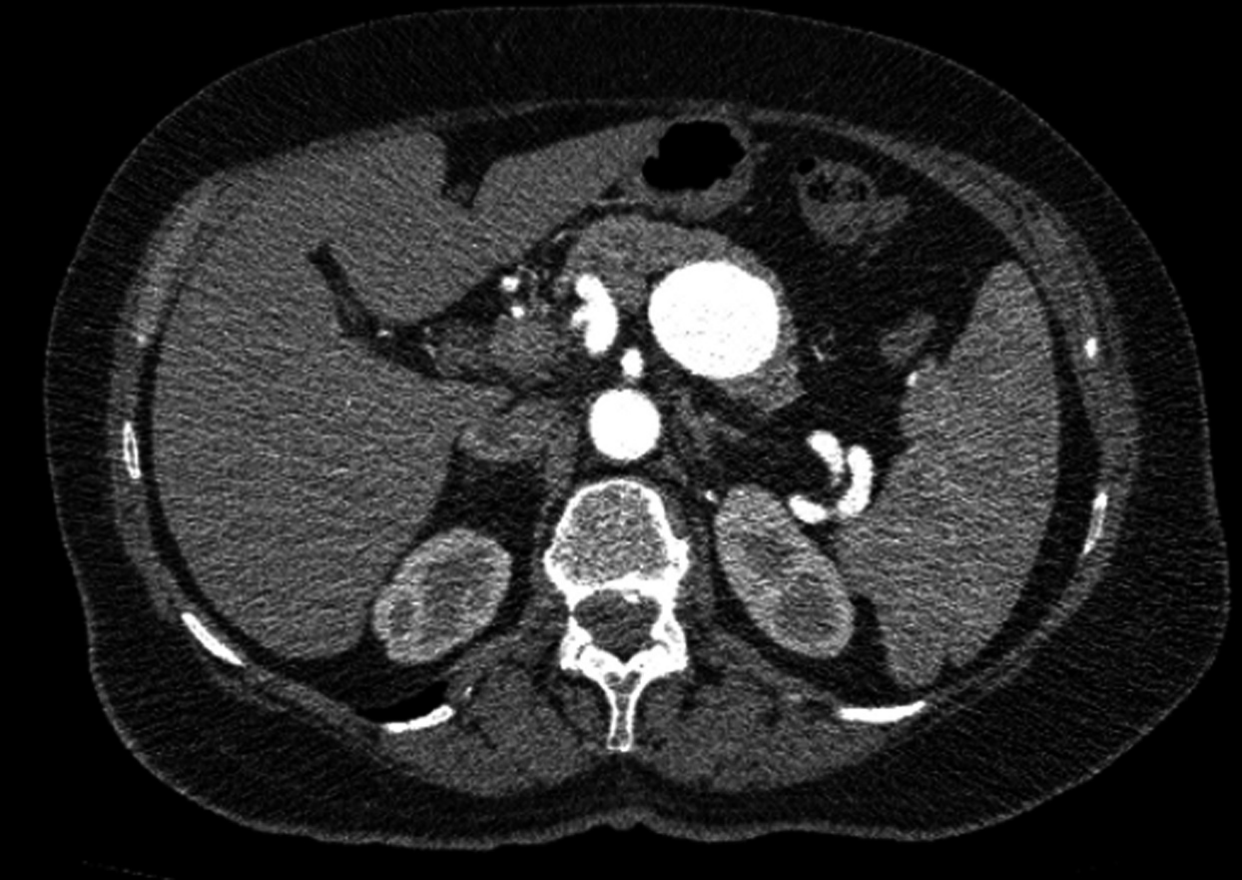
Abdominal CT angiography showing splenic artery aneurysm.

**Question 2**: What kind of surgical approach would you prefer to treat this patient?

**Answer 2**: The patient underwent laparotomy, and the exploratory findings revealed a saccular SAA that was tightly attached to the pancreas (Fig. 2A). Aneurysmectomy was therefore not feasible. The proximal and distal edges were ligated, and aneurysmotomy was performed. When the aneurysmal sac was opened, an aberrant vascular orifice was encountered, and suture ligation was performed. Following capitonnage of the sac, an end‐to‐end arterial anastomosis was successfully completed (Fig. 2B).

**Figure 2:**
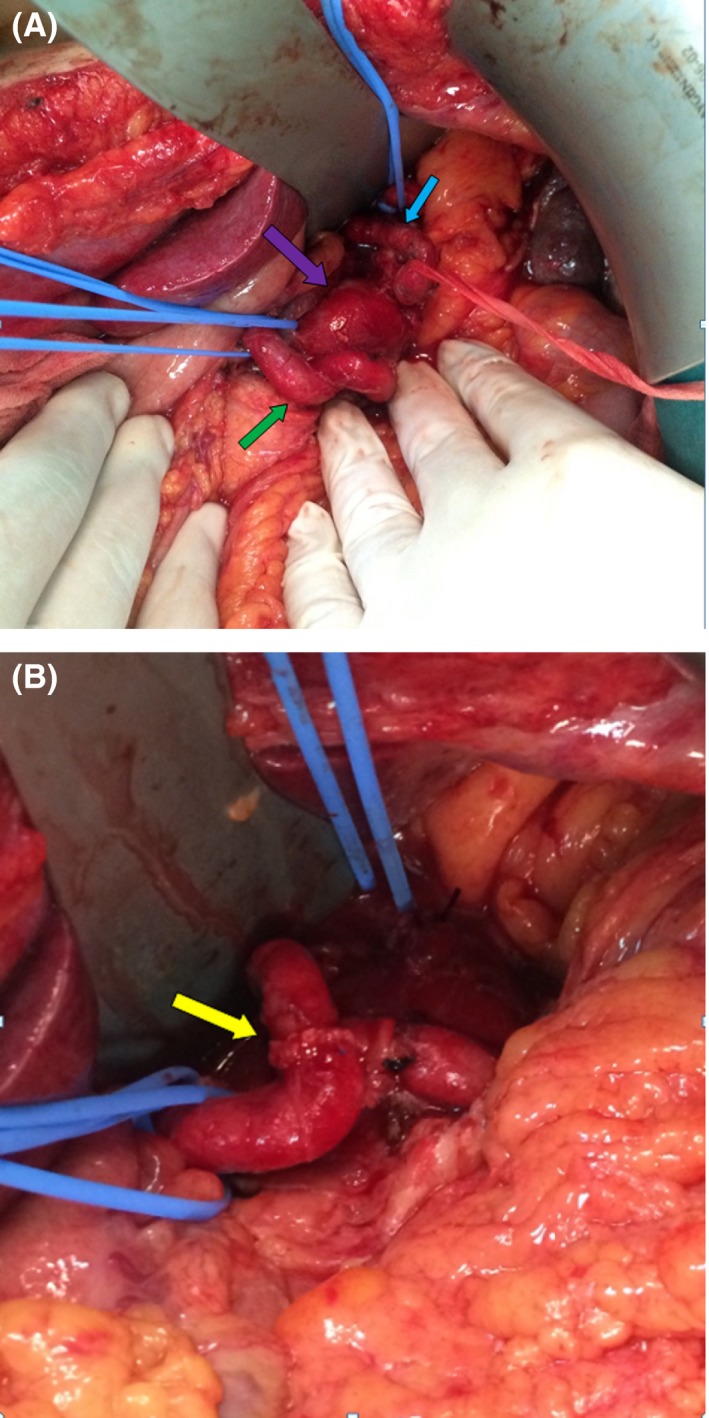
(A) Splenic artery aneurysm with purple arrow pointing to the sac, green arrow pointing to the proximal part of the artery, and blue arrow pointing to the distal part of the artery. (B) End‐to‐end arterial anastomosis indicated with yellow arrow.

Splenic hilar or distal splenic artery aneurysms are usually treated with aneurysmectomy with splenectomy. Proximal or fusiform middle‐third portion splenic artery aneurysms can be ligated directly or resected without the need of revascularization 1. However, as we detected an aberrant vascular orifice supplying the aneurismal sac in this patient, we suggest performing an aneurysmotomy and exploring the sac in all cases to rule out this phenomenon.

## Conflict of Interest

None deaclared.
